# Bismuth Oxyhalide-Based Materials (BiOX: X = Cl, Br, I) and Their Application in Photoelectrocatalytic Degradation of Organic Pollutants in Water: A Review

**DOI:** 10.3389/fchem.2022.900622

**Published:** 2022-07-11

**Authors:** G. Xavier Castillo-Cabrera, Patricio J. Espinoza-Montero, Paulina Alulema-Pullupaxi, José Ramón Mora, Milton H. Villacís-García

**Affiliations:** ^1^ Escuela de Ciencias Químicas, Pontificia Universidad Católica Del Ecuador, Quito, Ecuador; ^2^ Facultad de Ciencias Químicas, Universidad Central Del Ecuador, Quito, Ecuador; ^3^ Universidad San Francisco de Quito, Quito, Ecuador

**Keywords:** photoelectrocatalysis, bismuth oxyhalide, visible-light, organic pollutants, water treatment

## Abstract

An important target of photoelectrocatalysis (PEC) technology is the development of semiconductor-based photoelectrodes capable of absorbing solar energy (visible light) and promoting oxidation and reduction reactions. Bismuth oxyhalide-based materials BiOX (X = Cl, Br, and I) meet these requirements. Their crystalline structure, optical and electronic properties, and photocatalytic activity under visible light mean that these materials can be coupled to other semiconductors to develop novel heterostructures for photoelectrochemical degradation systems. This review provides a general overview of controlled BiOX powder synthesis methods, and discusses the optical and structural features of BiOX-based materials, focusing on heterojunction photoanodes. In addition, it summarizes the most recent applications in this field, particularly photoelectrochemical performance, experimental conditions and degradation efficiencies reported for some organic pollutants (e.g., pharmaceuticals, organic dyes, phenolic derivatives, etc.). Finally, as this review seeks to serve as a guide for the characteristics and various properties of these interesting semiconductors, it discusses future PEC-related challenges to explore.

## 1 Introduction

Water pollution is an environmental problem that has attracted worldwide attention in recent years. Contaminants of emerging concern (CECs)—such as the active compounds in pharmaceuticals (antibiotics, pain relievers, etc.), personal care product, endocrine disrupting compounds, agrochemicals (herbicides, insecticides, fungicides, etc.), dyes, detergents (surfactants), and disinfection byproducts—are persistent organic molecules that can be found in wastewater, treated water, and natural water sources in concentrations ranging from mg/L to μg/L (Quesada et al., 2019; Gao et al., 2021; Li et al., 2021; Martínez-Huitle et al., 2015; Sanganyado et al., 2017; Garcia-Segura et al., 2020; Vasseghian et al., 2021; Zhang et al., 2021). CECs are generally complex and chemically stable molecules, which makes the removal process or degradation by traditional methods very complicated (Arotiba, Orimolade and Koiki, 2020). Currently, the consumption of inadequately treated water represents a risk to human health, as globally, approximately 700 million people do not have access to drinking water (Urban, 2017).

An underexplored area of water contamination research involves the active pharmaceutical compounds that appear after being metabolized by the body. These compounds are discarded in the form of metabolites, which are persistent in bodies of water (Quesada et al., 2019). Also contributing to water pollution are synthetic organic dyes, which are discharged into natural effluents mainly from the textile and paper industries (Nguyen et al., 2020). Another source of serious water pollution comes from the agricultural industry’s indiscriminate use of agrochemicals, particularly synthetic pesticides, which, after being applied to plantations are transported by rainwater runoff to the main water bodies (Vasseghian et al., 2021). These organic pollutants remain in the environment and affect human health, because of their poor biodegradability and high levels of reactivity in the body, which leads to abnormal physiological processes (endocrine disruptors), enhanced bacteria resistance to antibiotics, and the appearance of certain types of cancer, as well as imbalanced chemical reactions in the body (Bagheri et al., 2017; Quesada et al., 2019). Therefore, the development of efficient technologies to completely remove or degrade stable organic pollutants is important. Several recent studies have reported relatively high effectiveness rates for the removal of organic contaminants in aqueous media, evaluating various techniques such as nanofiltration (Garcia-Ivars et al., 2017), ozonation (J. Wang & Bai, 2017), adsorption (Nguyen et al., 2020), photocatalysis (Quesada et al., 2019), and electrocatalysis (Singh and Goldsmith, 2020), the latter two being of special interest in the present review.

The development of photocatalysts for environmental remediation has been widely investigated, with emphasis on materials designed to harness solar energy (Hassani et al., 2021). However, a major disadvantage of photocatalysts is their photocatalytic efficiency, which is directly related to their intrinsic stability. On the other hand, photoelectrocatalysis (PEC) is an alternative that can substantially improve the limitations of photocatalytic processes. PEC consists of shining light on a semiconductor film, placed on a conductive substrate (photoelectrode), with a sufficient energy to overcome the energy barrier of the semiconductor band-gap (hν ≥ E_g_). This energy promotes the formation of charge carriers; electrons (e^−^) migrate from the valence band towards the conduction band ( ${\text{e}}_{\text{CB}}^{-}$ ), leaving empty energetic states of electrons called “holes” (h^+^) in the valence band ( ${\text{h}}_{\text{VB}}^{+}$ ). Simultaneously, a potential perturbation is applied to prevent the recombination of electron/hole pairs ( ${\text{e}}_{\text{CB}}^{-}$ / ${\text{h}}_{\text{VB}}^{+}$ ), which increase the availability of $h_{VB}^{+}$ that promotes the generation of hydroxyl radicals (^•^OH) by water oxidation. The ^•^OH is the second strongest oxidant that exists in nature, and is responsible for oxidizing persistent organic matter, even to its total mineralization, Figure 7 (Mousset and Dionysiou, 2020; Alulema-Pullupaxi, et al., 2021a; Wang et al., 2021; Wei et al., 2021). The most widely studied semiconductor material in this field is TiO_2_ (Xu et al., 2019; Alulema-Pullupaxi et al., 2021b; Sigcha-Pallo et al., 2022). However, due to its approximate band-gap energy of 3.2 eV, its application in photoelectrochemical processes with sunlight is limited, as this energy value corresponds to the UV range of the electromagnetic spectrum, which results in limited peak power conversion. Therefore, interest in the development of new visible light active semiconductor materials with potential photoelectrocatalytic applications has grown in recent years. Of these materials, bismuth oxyhalides (BiOXs) stand out as novel materials for photocatalytic and photoelectrocatalytic applications (Gao et al., 2021).

BiOXs are p-type semiconductors, meaning the energetic arrangement of their conduction and valence bands means favor reduction processes over oxidation. Pristine BiOXs (X = Cl, Br, I) are layered materials with an open crystal structure, exhibiting properties such as easy modification and formation of heterogeneous structures with other materials, chemical stability and ability to absorb UV and visible light because their narrow band-gaps (Gao et al., 2021; Wei et al., 2021). Due to these properties, when BiOXs couple with other semiconductors, the generated synergistic effect modifies their band structure and leads to oxidation processes, which is of particular interest for pollutant degradation. In this context, BiOX-based materials exhibit attractive properties for PEC. However, despite numerous studies on the development of BiOX-based photocatalysts, only a few of these materials have been applied in the degradation of organic pollutants through PEC (Li et al., 2021).

Based on the above, this review provides an overview of controlled BiOX powder synthesis methods, heterojunction formation and charge transfer mechanisms at the interface and the structural and optical properties of BiOX-based materials. Additionally, the most recent research on the photoelectrocatalytic degradation of organic pollutants is summarized, and future challenges in the development of semiconductor materials in PEC for environmental remediation are discussed.

## 2 BiOXs (X = Cl, Br, I) Synthesis Methods

The controlled synthesis methods used to produce BiOXs depend on the desired structure and morphology. The following is an overview of reported BiOXs powder synthesis methods for different applications.

### 2.1 Hydrothermal/Solvothermal Method

The hydrothermal/solvothermal method is a soft chemical method using different solvents (hydrothermal: water; solvothermal: other solvents), in which pressure is generated to promote chemical reactions, increasing the reactivity and precursors solubility (Wei et al., 2021). The most common source of Bi using this method is bismuth nitrate pentahydrate (Bi(NO_3_)_3_·5H_2_O), from which various micro and nanostructures with different BiOX compositions can be synthesized (Gao et al., 2021). For example, Xi Zhang et al. (2008) synthesized BiOXs powders (X = Cl, Br, and I) by stirring a mixture containing Bi(NO_3_)_3_·5H_2_O dissolved in ethylene glycol and inorganic salts of the halogens (KCl, NaBr, and KI) for 30 min; after autoclave heating at 160°C under self-generated pressure, the precipitates were collected, washed, and dried at 50°C. Thus, by controlling the kinetic and thermodynamic parameters of the reaction, the semiconductors’ morphology, crystalline phase, size, and composition can be adjusted. This method is versatile, as various suitable solvent can be used for synthesis, the choice of solvent not only determines the morphological characteristics of BiOXs, but also allows control of the particle size during crystal growth.

### 2.2 Hydrolysis Method

Unlike the hydro/solvothermal method, in synthesis *via* hydrolysis, several bismuth salts can be used as precursors, including halides, nitrates, and oxides. However, controlling the reaction to obtain dimensionally uniform products is more complicated, even when the reaction conditions are not severe (Wei et al., 2021). The precursor compounds, greatly impact the morphology and yield in the synthesis of these catalysts. Additionally, the solvent affects crystal growth, which is an important factor in photocatalytic performance (Li et al., 2021). Using direct hydrolysis, Su et al. (2014) synthesized bismuth oxyiodide (BiOI) with a hierarchical flower-like structure with BiI_3_ as a precursor. The hydrolysis method is suitable to prepare photoelectrodes using electrodeposition techniques, and the reaction medium also serves as an electrochemical medium for electrode modification. Electrodepositions with BiOX have been tested especially on fluorine-doped tin oxide (FTO) and indium tin oxide (ITO) electrodes (Ling et al., 2020).

### 2.3 Calcination Method

**FIGURE 1:**
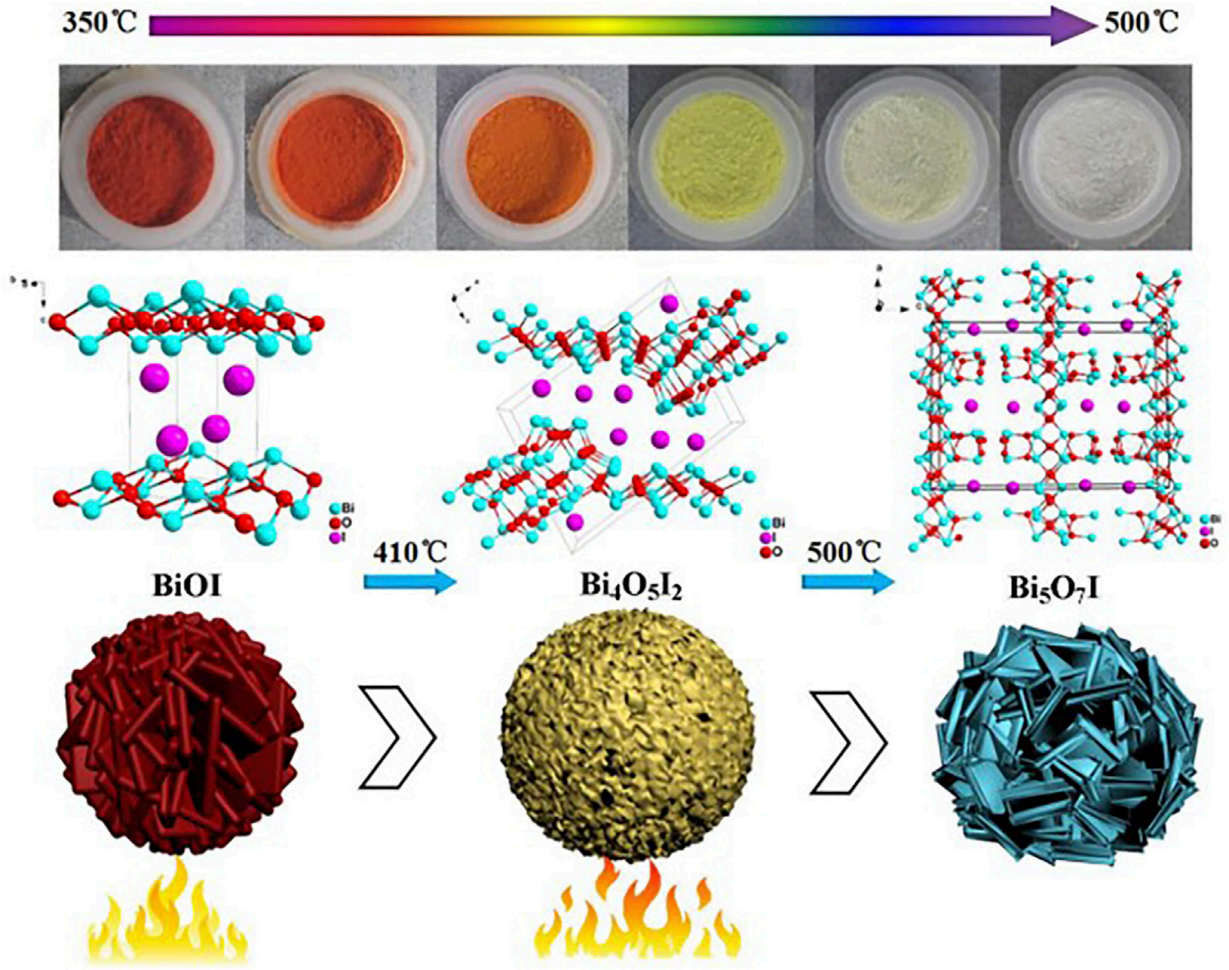
Calcination synthesis of Bi_4_O_5_I_2_ and Bi_5_O_7_I composites at different temperatures. From reference Huang et al. (2017).

Calcination is a useful method when a phase transformation of the bismuth oxyhalide to a bismuth-rich bismuth oxyhalide is desired, as heating removes unstable halogen atoms in the crystal structure (Gao et al., 2021; Wei et al., 2021). Using this method, Huang et al. (2017) synthesized Bi_4_O_5_I_2_, Bi_5_O_7_I, and Bi_4_O_5_I_2_-Bi_5_O_7_I composites using BiOI as the precursor (Figure 1). Calcination temperatures were set at 350, 380, 410, 440, 470, and 500°C. After heating, the samples were collected and characterized by *x*-ray diffraction (XRD); the diffraction patterns indicated the phase transformation of BiOI. In another study, Lee et al. (2015) described the synthesis of some bismuth-rich BiOI-based composites via hydrothermal methods without the need for calcination.

### 2.4 Ultrasound-Assisted Method

Ultrasound has been used to complement to traditional methods of producing BiOX-based composites, such as the spray-assisted method. Using a hydrothermal method, Qin et al. (2020) fabricated a BiOI/Bi_2_O_4_ Z-type heterojunction by subjecting different amounts of Bi_2_O_4_ (input power of 300 W and a frequency of 40 kHz) to ultrasound for 1 hour. Morphology results of this material showed that the Bi_2_O_4_ structure was irregular and consisted of nanorods of various sizes. Meanwhile, the structure of BiOI was hierarchical with agglomerations of flower-like nanosheets. This composite exhibited excellent visible light properties for the degradation of Rhodamine B (RhB) in a photocatalytic system.

**FIGURE 2:**
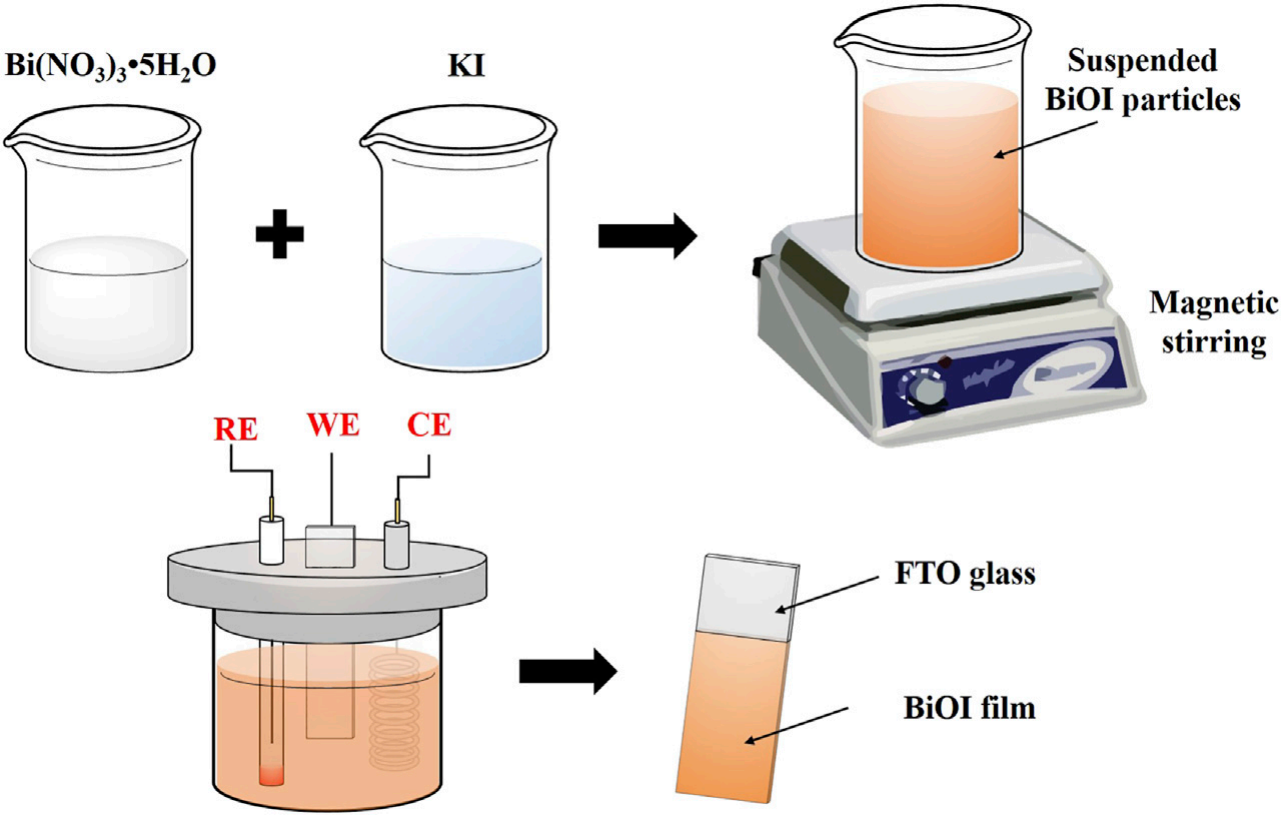
Fabrication of a BiOI-modified FTO glass electrode using cathodic electrodeposition.

In photoelectrocatalytic processes, the design of a stable and functional photoelectrode based on a suitable semiconductor is important. Potentiostatic cathodic electrodeposition is one of the most common techniques to create BiOX-modified electrodes. In a typical three-electrode system, the working electrode (commonly FTO or ITO), the reference electrode and the counter electrode are placed in a reaction medium, usually a hydrolysis synthesis medium. (Ye et al., 2015; Orimolade and Arotiba, 2022). A suitable potential difference is applied at a specific time (depending on the material), and the BiOX particles migrate to the electrode and deposit on its surface, covering it with a thin film. The coupling of BiOX with the electrode (another semiconductor) creates the so-called BiOX-based material, which has a potential role in electrochemical, photochemical, and photoelectrochemical processes. Figure 2 presents a simple schematic of the electrodeposition of BiOI on a typical FTO electrode. Other methods to produce photoelectrodes have been successfully employed, such as the SILAR method (Ma et al., 2021), chemical bath deposition (Zhang et al., 2020), and the dipping process (Cong et al., 2017).

## 3 BiOX-Based Materials

**FIGURE 3:**
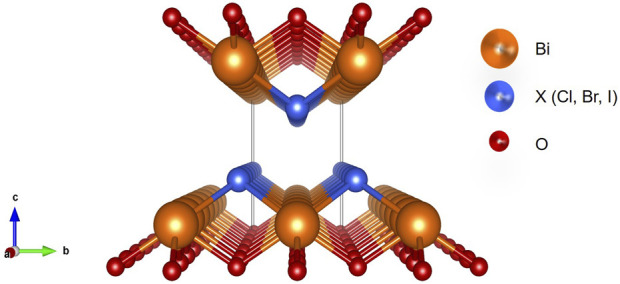
Crystal structure of bismuth oxyhalide (BiOX, X = Cl, Br, and I). Adapted from (Ganose et al., 2016).

BiOXs belong to the class of ternary semiconducting materials of group V-VI-VII; they crystallize in a tetragonal matlockite structure with a perfect (001) facet (Gao et al., 2021; Wei et al., 2021). As illustrated in Figure 3, these halogen-modified bismuth oxides are layered materials, that is, they consist of [Bi_2_O_2_]^2+^ layers successively intercalated with double layers of halide X^−^ ions linked by Van der Waals interactions (Ganose et al., 2016). This layered structure gives them an intrinsic stability, since a space is formed between layers, which polarizes the atoms and orbitals to obtain a dipole that can separate the photogenerated charges and prevent their recombination. This last feature is of great interest in both photocatalytic and photoelectrocatalytic processes (Zhao et al., 2012).

**FIGURE 4:**
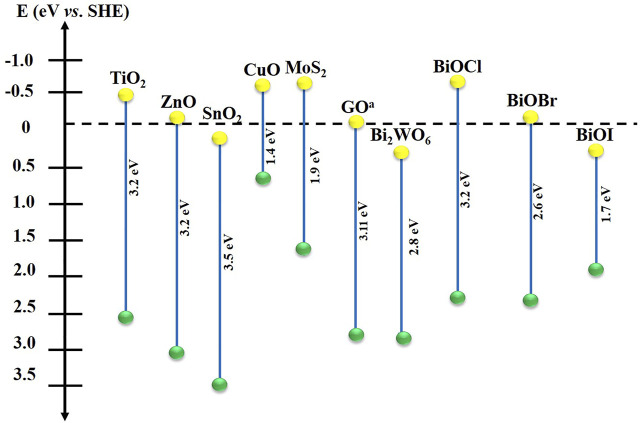
Band-gap of various semiconductor materials. Valence band (green); conduction band (yellow). ^a^ Graphene oxide. Adapted from: (Kumar et al., 2021; Olea, Bueno and Pérez, 2021).

Among the most widely used semiconductor materials in PEC, TiO_2_ is the mostly known, due to its stability, friendliness, non-toxicity, and wide availability (Olea et al., 2021). However, TiO_2_ has a wide band-gap potential (∼3.2 eV vs. NHE), which limits its applications in PEC. To compensate for this disadvantage, some photocatalysts have narrower band-gaps, which can absorb the visible light spectrum and limit the recombination of the photogenerated charge carriers (Figure 4). It is relevant to note that most of these semiconductor materials are generally used in the production of photoanodes (for oxidation reactions). These materials belong to the n-type semiconductors, where the conduction band is relatively full of electrons that are then transported by an external circuit in photoelectrocatalytic processes. Additionally, the photogenerated holes must have a sufficient anodic potential to produce ^•^OH when they react with the aqueous medium (Olea et al, 2021). On the other hand, p-type semiconductors such as CuO and pristine BiOX (Ye et al., 2015), have valence band relatively full of positive vacancies (h^+^), these materials are used for reduction reactions (photocathode production) (Kusmierek, 2020; Olea et al., 2021). Thus BiOX-based photoanodes have been designed to improve the optical and performance properties of n-type semiconductors, including TiO_2_ and various others (Figure 4).

To overcome the limitations of n-type semiconductors in photocatalytic and photoelectrocatalytic processes, the formation of heterojunctions with BiOXs has been considered an excellent option. The formation of heterostructures results in a strategy to carrier separation and decrease the recombination rate (Li et al., 2020). Heterojunctions result from the interaction between two semiconductors with different band-gaps. The Fermi levels of both semiconductors align at the interface, and the conduction and valence bands bend toward different energy levels (Kumar et al., 2021; Olea et al, 2021). Heterojunctions can be classified by the charge transfer mechanism at the interface or by the types of semiconductors that form the heterojunction. The p-n type heterojunction formed by the coupling of an n-type semiconductor and a p-type semiconductor is in agreement with the type II mechanism (Ye et al., 2015).

**FIGURE 5:**
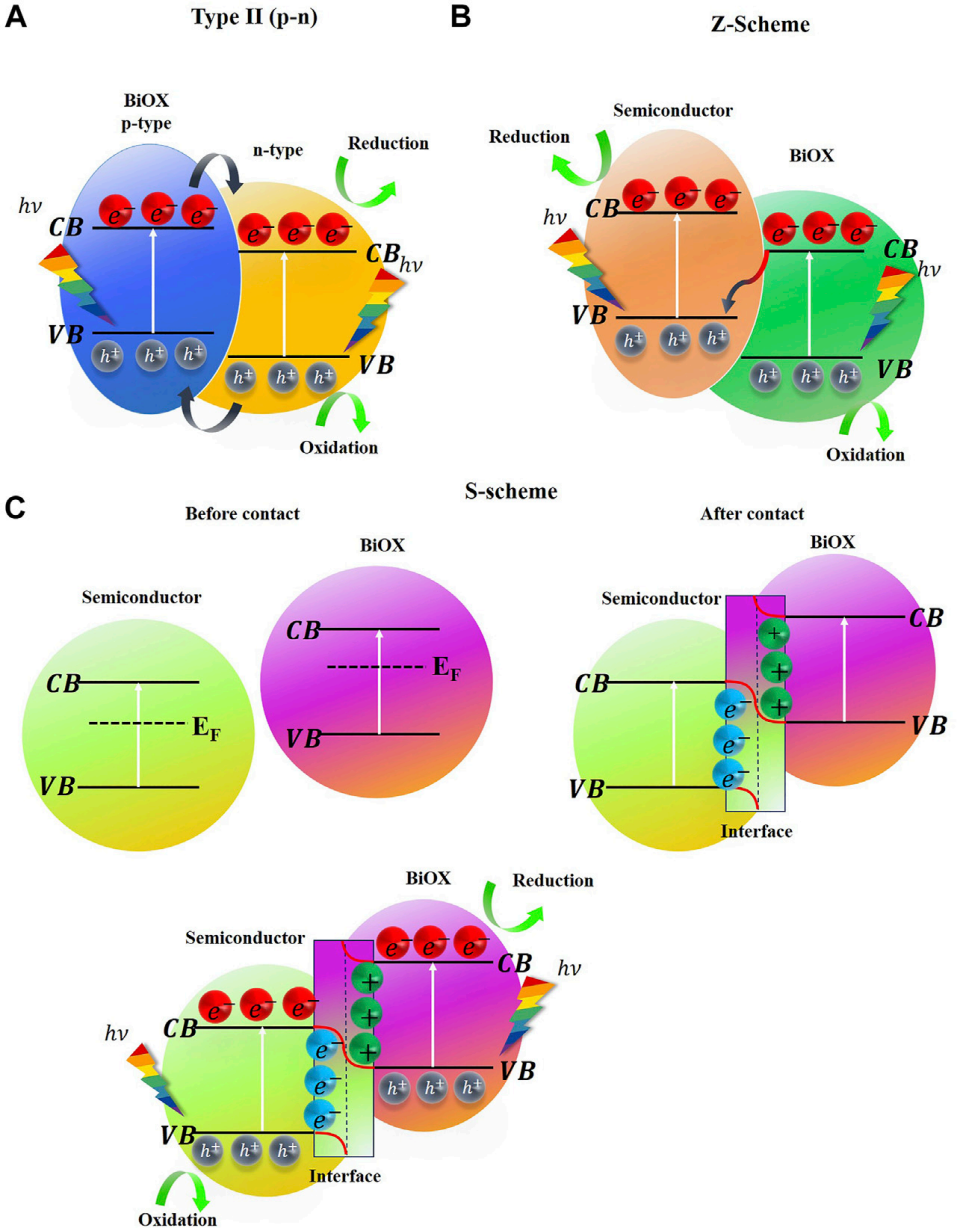
**(A)** Heterojunction p-n (type II mechanism), **(B)** Z-scheme mechanism, **(C)** S-scheme mechanism. CB and VB are the conduction and valence bands, respectively. E_F_ Fermi level.

In this regard, Guo et al. (2020) fabricated a Bi_2_WO_6_/BiOCl heterostructure using a solvothermal method with excellent photochemical performance. The separation and charge transfer along the interface between the semiconductors showed that electrons flowed from Bi_2_WO_6_ to BiOI according to the type II mechanism (Figure 5A), as modeled by electron difference density. In addition, the internal electric field of BiOCl further enhances charge separation. Similar results for the Bi_2_WO_6_/BiOBr heterojunction synthesized using a two-step ionic liquid-assisted method showed that charge transfer occurred, evidenced by the irregular BiOBr structure adhering to the flower-like morphology of Bi_2_WO_6_ (Pancielejko et al., 2021). Another heterostructure modification with BiOX has involved composites incorporated with carbonaceous materials. Due to their hierarchical structures and large active areas, the Bi-C bonds increase the speed of electron mobility between the bands (Hou et al., 2017). For example, Rashid et al. (2020) used a simple hydrothermal method to synthesize a BiOCl/GO composite (GO: graphene oxide). As shown in Figure 4, BiOCl is not active against visible light; however, by adding GO, the band-gap shifted towards 3.08 eV, which promote catalytic reactions in the medium even under sunlight irradiation. Although BiOX-based composites appear to improve charge transport at the heterojunction interface, the incorporation of defects in BiOXs’ crystal structure has been examined, and results have shown promising applications in other fields of advanced oxidation processes (Shi et al., 2021).

Another type of BiOX-based heterojunction is the Z-scheme (Figure 5B), in which only electrons from the conduction band of the n-type semiconductor are directed toward the valence band of the p-type semiconductor (Y. Li et al., 2021). The type of heterojunction produced depends on the involved the photocatalysts’ band structure, their electronic nature, and the potential levels at which the reactions occur. In this context, Qin et al. (2020) designed a BiOI/Bi_2_O_4_ heterojunction using an ultrasound-assisted hydrothermal method. Because the valence band of BiOI was more positive than the reduction potential of ^−^OH/^•^OH and the conduction band of Bi_2_O_4_ was more negative than the oxidation potential of O_2_/O_2_^•−^, the possible mechanism for charge transport consisted of electrons from the conduction band of BiOI migrate to the valence band of Bi_2_O_4_ to neutralize the photoexcited holes (Z-scheme). Another effective heterojunction charge transfer mechanism is the S-scheme (Figure 5C). Herein, two semiconductors—one dedicated to oxidation reactions (more positive valence band) and one reduction semiconductor (more negative conduction band)—come together and generate an interface with a strong redox potential; this is because, the photoexcited electrons are kept in the conduction band of the reduction photocatalyst and the holes are kept in the valence band of the oxidation photocatalyst (Xu et al., 2020). In addition, a spontaneous diffusion of the remaining electrons and holes from the reducing semiconductor to the oxidizing semiconductor and vice versa occurs, causing the bands to bend in the interface to maintain Fermi level equilibrium (Liao et al., 2021). Related studies have examined the S-scheme heterojunction of the ternary composite Bi_7_O_9_I_3_/g-C_3_N_4_/Bi_3_O_4_Cl synthesized via the oil bath method (Yuan et al., 2022). This composite has a double interface between the oxyhalides and g-C_3_N_4_. Electrons migrate from g-C_3_N_4_ towards Bi_7_O_9_I_3_ and Bi_3_O_4_Cl simultaneously, causing band bending up and down the interface. This result in charge transfer and recombination blocking solely at the interface, leaving only electrons and holes with strong redox capabilities for light promoted reactions (Yuan et al., 2022). In this and other cases g-C_3_N_4_ acts as a kind of charge carrier at the conducting interface (Yan, Liu and Jin, 2021), strengthening the internal electric field and preventing recombination when light acts on the photocatalysts. This also includes quaternary BiOX-based composites, such as BiO_x_Cl_y_/BiO_m_Br_n_/BiO_p_I_q_/g-C_3_N_4_ (Lin et al., 2022). Further, a heterostructure formed by coupling a noble metal with a semiconductor forms a typical Schottky junction (Bai et al., 2015), wherein the metal concentrates the photogenerated holes of the p-type semiconductor (BiOX). In this way, a so-called Schottky barrier is formed which prevents the return of charge carriers from the metal back to the semiconductor, that is, the charge flow occurs unidirectionally (Bai et al., 2015; Kumar et al., 2021). In this context, Orimolade and Arotiba, (2022) fabricated a functional BiOI photoelectrode decorated with Ag nanoparticles supported on FTO glass. By loading silver particles on the BiOI/FTO electrode, the photocurrent responses increased. This is attributed to Ag acting as a photosensitizer by absorbing photons and controlling direct electron transfer.

### 3.1 Band Structure

Theoretical studies based on *ab initio* calculations can successfully predict some electronic, optical and structural properties of these materials (BiOX). Some of these properties are related to the formation of continuous bands of energy levels (atomic orbitals). One of the most important advantages of semiconductor materials over other photocatalysts is their narrow band-gap energy. Poznyak and Kulak, (1990) were the first to analyze the photoelectrochemical properties of BiOX using an anodic bismuth oxidation method; they synthesized BiOX films (X = Cl, Br, and I), finding band-gap values of 3.50 eV (BiOCl), 2.92 eV (BiOBr) and 1.90 eV (BiOI). These band-gap energy values have been confirmed by computational calculations employing density functional theory (DFT) (Zhao et al., 2012). The valence band maximum of BiOX consists of np orbitals of the halogens (n = 3, 4, and 5 for Cl, Br, and I, respectively) and 2p orbitals of oxygen, while the conduction band minimum contains 6p orbitals of bismuth (Zhao et al., 2012). Recently, Barhoumi and Said (2021) theoretically examined the band structures of BiOXs, including BiOF, using the generalized gradient approximation (GGA) and GW approximation (GWA), supported by density functional theory (DFT), obtaining band-gap results more in line with those reported experimentally, from 2.33 eV for BiOI to 4.04 eV for BiOF.

### 3.2 Crystal Structure and Optical Properties

Although BiOXs exhibit narrow band-gap energies and good activity under visible light, their low quantum efficiency limits their applications in both photocatalysis and PEC because of the high recombination rate of photogenerated ${\text{e}}_{\text{CB}}^{-}/{\text{h}}_{\text{VB}}^{+}$ pairs (Cao et al., 2018; Chen et al., 2018). To improve their optical properties and reduce charge recombination, doping, surface modification, and heterojunction formation have been adopted as alternatives to accelerate charge transfer and increase efficiency in PEC degradation applications (Li et al., 2021; Wang et al., 2021). In recent years, many studies have been reported discussing the optical and structural properties of BiOX-based materials from a DFT-based theoretical perspective, including indium-doped BiOX (Li et al., 2018), MoS_2_/BiOX (X = Cl, Br, and I) heterojunctions (Gao et al., 2019), BiOCl/BiOBr (Cui et al., 2019; Li et al., 2021), and stoichiometric low-index BiOI surfaces (Dai and Zhao, 2017).

#### 3.2.1 Crystalline Phase and Morphology

**FIGURE 6:**
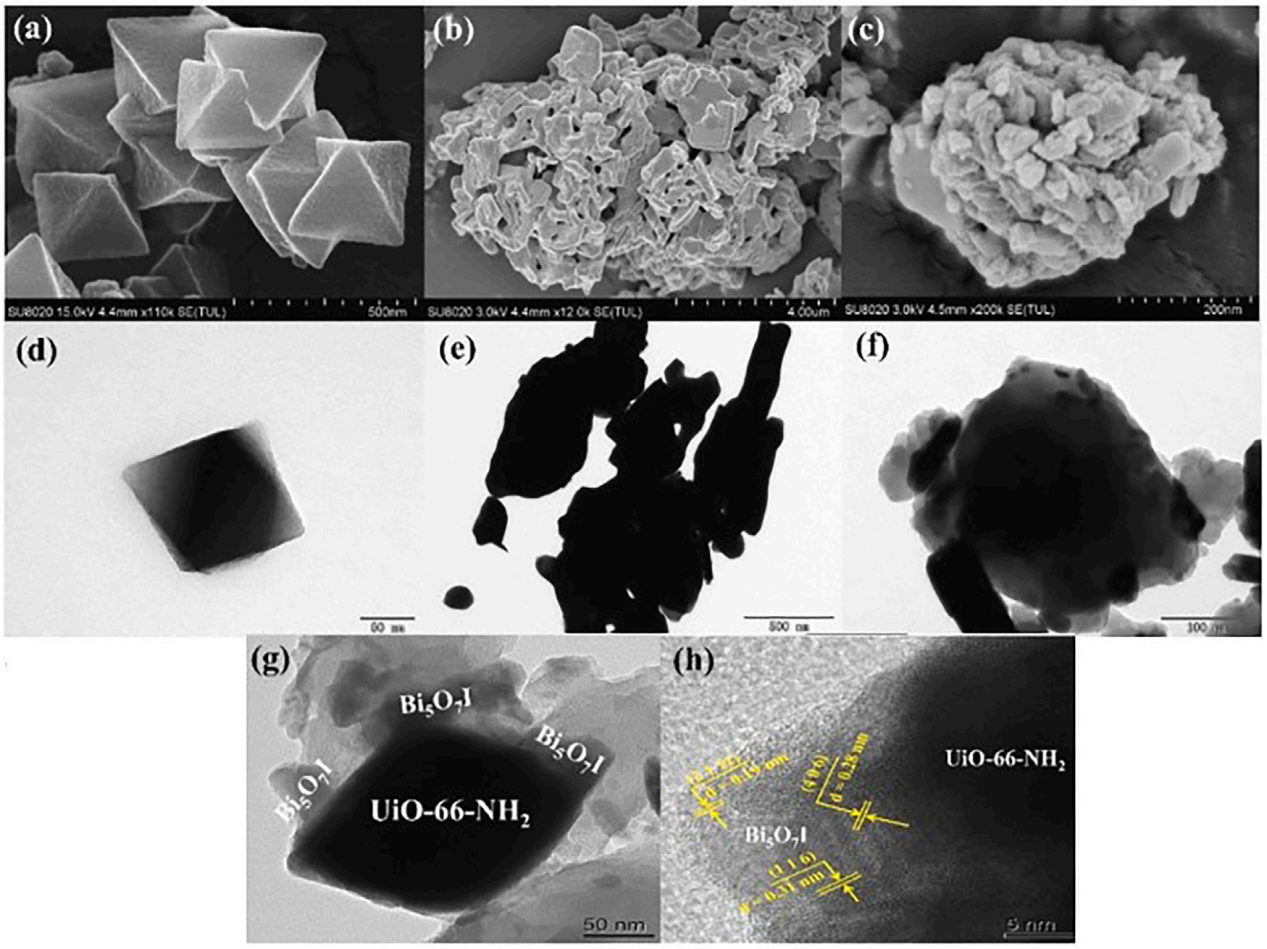
**(A,D)** SEM and TEM micrographs of UiO-66-NH_2_, **(B,E)** Bi_5_O_7_I, **(C,F)** BU-5, and **(G,H)** HRTEM. From reference (Zhao et al., 2021).

The stability of BiOXs is mainly due to their layered structure, a feature that promotes the formation of new structures with other semiconducting materials. Q. Wang et al. (2019) prepared a Bi-modified BiOI-Bi_2_O_3_ heterojunction using an *in situ* reduction method applying UV light with BiOI-Bi_2_O_3_ previously prepared by single immersion at room temperature method reported by Cong et al. (2017). Using high-resolution transmission electron microscopy (HRTEM), the morphology of the Bi/BiOI-Bi_2_O_3_ film was investigated; results, showed that the Bi^0^ (012) planes and BiOI (110) planes were predominant, where Bi^0^ was in direct contact with BiOI. On the other hand, Xu et al. (2020) synthesized stoichiometric BiOCl_x_I_1-x_ (x = 1.0, 0.75, 0.5, and 0.25) using an alcoholysis method at room temperature. XRD showed that the percentage exposure of the (001) crystalline plane (matlockite) was predominant in the BiOCl_x_I_1-x_ solid solutions. Moreover, when the stoichiometric value x decreased, the crystalline parameters *a* and *c* of BiOCl_x_I_1-x_ tended to increase, because the interlayer spacing of the crystal structure expanded as I atoms replaced Cl atoms. Using field emission scanning electron microscopy (FESEM), they found that the synthesized BiOCl_x_I_1-x_ materials exhibited a two-dimensional flake-like structure, which when stacked irregularly formed microsphere or flower-like architectures. Furthermore, C. Zhao et al. (2021) successfully prepared a Bi_5_O_7_I/UiO-66-NH_2_ (UiO-66-NH_2_ is a zirconium organometallic) heterojunction through a ball milling method. Bi_5_O_7_I synthesized using a calcination method of BiOI prepared by co-precipitation was characterized by XRD; Bi_5_O_7_I exhibited high crystallinity. Scanning electron microscopy (SEM), transmission electron microscopy (TEM) and HRTEM results showed that Bi_5_O_7_I had a three-dimensional coral-like structure built by nanorods that formed agglomerations (Figure 6). Additionally, the microscopy results suggested that the closed interaction of the Bi_5_O_7_I/UiO-66-NH_2_ heterojunction promoted the transfer of photogenerated charges, thus increasing the rate of catalytic reactions. In another study, Pancielejko et al. (2021) prepared a Bi_2_WO_6_/BiOBr heterojunction based on a two-step synthesis. Ultraviolet-visible diffuse reflectance spectroscopy (UV-vis DRS) results showed that increasing the amount of BiOBr the response to visible light is decreased, because Bi_2_WO_6_ surface was completely covered, causing light shielding. Thus, the synthesis method plays an important role in the structure, morphology, and stoichiometry of the desired BiOX. Many photocatalysts are limited by their supporting material, since physical interaction with their surface would modify their morphology. As BiOX are layered materials, a semiconducting surface with a large number of irregular interstices could affect the stability of the resulting material.

#### 3.2.2 Optical Properties

The ability of BiOXs to absorb light in the visible spectrum plays a key role in their photocatalytic activity (Wilczewska et al., 2021). The success of BiOX-based materials in photocatalytic and photoelectrocatalytic applications is related to the hierarchy of their surface structures, which significantly improve the ability to absorb visible light with respect to BiOXs. In this regard, using an *in situ* chemical deposition-precipitation method, Wang et al. (2020) synthesized a 2D/2D BiOBr/Bi_12_O_17_Cl_12_ heterojunction and evaluated its photocatalytic ability regarding the degradation of pollutants in water. Based on Tauc plots, they obtained a band-gap energy of 2.28 eV for the BiOBr/Bi_12_O_17_Cl_12_ composite. Additionally, they investigated the light absorption ability with UV—vis DRS and observed the two-dimensional structure of BiOBr/Bi_12_O_17_Cl_12_ exhibited a significant enhancement in visible light absorption, as the absorption edge was red-shifted by approximately 530 nm, whereas pure BiOBr exhibited poor absorption (∼430 nm of the absorption edge). Similar results previously reported by Lee et al. (2019) for a t-PbBiO_2_I/Bi_5_O_7_I/g-C_3_N_4_ composite prepared using hydrothermal processes, showed that introducing g-C_3_N_4_ into the t-PbBiO_2_I/Bi_5_O_7_I structure improved the visible light response and efficiently promoted photogenerated charge carriers. Likewise, Wu et al. (2019) developed BiOCl/Mo_2_S composites using ultrasound-assisted synthesis, the interfacial formation of Bi-S bonds, charge separation and significant reduction of band-gap energy were promoted due to the formation of oxygen vacancies. Oxygen vacancy refers to a type of defect in semiconductor oxides (Huang et al., 2014). The band-gap energy of the composites was located between 3.45 and 3.03 eV and decreased as MoS_2_ mass ratio increased. Furthermore, using an *in situ* synthesis method, Qi et al. (2021) prepared a heterogeneous WS_2_/Mo_2_S/BiOCl structure and showed that by increasing the stoichiometric ratio of WS_2_/Mo_2_S, the light absorption band edge increased while BiOCl’s activity against light was enhanced. In a similar study, Liu et al. (2021) synthesized S-doped BiOBr (S-BiOBr) using a solvothermal method; DRS showed that the 0.5S-BiOBr (0.5S refers to sulfur mass ratio) doping exhibited high visible light trapping ability compared to BiOBr. The corresponding band-gap values were 2.85 eV for 0.5S-BiOBr and 3.00 eV for pure BiOBr. In addition, they showed that sulfur doping boosted the formation of oxygen vacancies, which promoted photogenerated charge transfer. Zhang et al. (2020) prepared a Z-type BiOX (X = Cl and Br)-Au-CdS heterojunction by chemical bath deposition. According to the UV-vis DRS spectra, the composite exhibited a slight red shift with respect to pure BiOX, this attributed to the strong interaction between Au and CdS. Additionally, photoluminescence spectroscopy results showed that the heterojunction presented a peak with lower intensity, which translates into a reduction of the recombination rate of the photogenerated ${\text{e}}_{\text{CB}}^{-}/{\text{h}}_{\text{VB}}^{+}$ pairs.

## 4 BiOX Applied in Photoelectrocatalysis

### 4.1 Principles of PEC

PEC constitute an advanced oxidation process (Kusmierek, 2020; Alulema-Pullupaxi et al., 2021a) and is considered an innovative technology, because it is capable of improving photocatalysis process. It has been used to decontaminate aqueous media where organic pollutants predominate in low concentrations and whose degradation or removal is not achieved with traditional processes. The photoelectrocatalytic mechanism is similar to photocatalysis, except that in PEC, a semiconductor material (photocatalyst) is deposited on the surface of an electrode to form a photoelectrode, whose electronic characteristics determine if it is a photoanode (n-type), where oxidative reactions could be carried out, or a photocathode (p-type), which favors reduction processes (Kusmierek, 2020; Olea, Bueno and Pérez, 2021). In addition, the semiconductor type, solution medium, pH value, nature of the supporting electrolyte, applied current, and light source are some of the factors that affect the efficiency of photoelectrocatalytic processes (Kusmierek, 2020).

**FIGURE 7:**
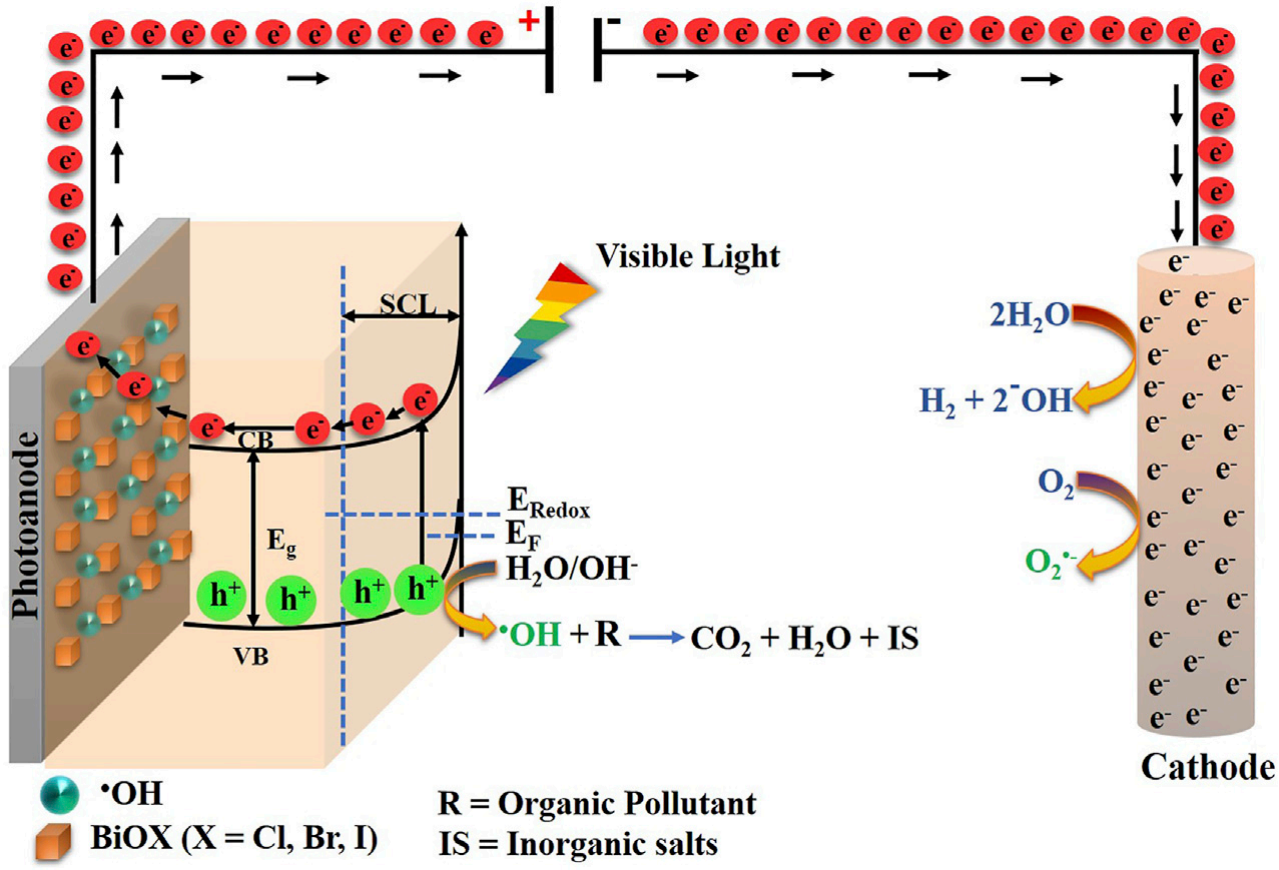
Photoelectrocatalytic mechanism using BiOX as semiconductor material. (SCL)-Space Charge Layer; (CB)-Conduction Band; (VB)-Valence Band; (E_g_) Band-gap energy; (e^−^) Conduction band electrons; (h^+^) Valence band holes; (E_F_) Fermi energy level.

Once the photoelectrode of interest has been selected, the photoelectrocatalytic process starts with the photoexcitation of electrons from the valence band (VB) to the conduction band (CB) of the semiconductor material. The energy difference between these two discrete energy levels is called the band-gap (BG or E_g_). The energy of the photons absorbed by the semiconductor material must be greater or equal to the band-gap energy $\;\left(\text{hν}\geq {\text{E}}_{\text{g}}\right)$ , to promote the formation of charge carriers, namely electrons in the conduction band $\left({\text{e}}_{\text{CB}}^{-}\right)$ and holes or positive vacancies in the valence band $\;\left({\text{h}}_{\text{VB}}^{+}\right)$ . These ${\text{e}}_{\text{CB}}^{-}/{\text{h}}_{\text{VB}}^{+}$ pairs are important in both photocatalysis and photoelectrocatalytic technologies (Tugaoen et al., 2017; Kusmierek, 2020; Alulema-Pullupaxi et al., 2021a; Alulema-Pullupaxi et al., 2021b; Olea et al., 2021; Sigcha-Pallo et al., 2022). Compared to heterogeneous photocatalysis, PEC also requires an applied bias potential; capable of extracting and transporting electrons through an external circuit, to avoid the recombination of the photogenerated ${\text{e}}_{\text{CB}}^{-}/{\text{h}}_{\text{VB}}^{+}$ pairs. This phenomenon is a typical disadvantage in photocatalysis (Tugaoen et al., 2017; Vargas et al., 2019; Kusmierek, 2020; Monllor-Satoca et al., 2020). Thus, successful PEC forms ^•^OH on the photoanode surface, which are capable of reacting with the target pollutant molecules and converting them into CO_2_, H_2_O, and inorganic salts (mineralization) or, in certain cases forming of simpler and readily biodegradable substances (Olea et al, 2021) (Figure 7). The photochemical mechanism is summarized below, with emphasis on the use of BiOX—based material (BiOX—BM) as semiconductor material, which are of interest in this review.

$$\text{BiOX}-\text{BM}+\;h\nu \to \text{BiOX}—\text{BM}\left({\text{e}}_{\text{CB}}^{-}+{\text{ h}}_{\text{VB}}^{+}\right)h\nu \geq {\text{E}}_{\text{g}}$$ (1)

$${\text{h}}_{\text{VB}}^{+}+{\text{H}}_{2}\text{O}/{\text{OH}}^{-}\to {\;}^{•}\text{O}\text{H}+{\text{H}}^{+}+{\text{e}}^{-}$$ (2)

$$\;{\text{e}}_{\text{CB}}^{-}+{\text{O}}_{2}\to {\text{O}}_{2}^{-•}$$ (3)

Reaction (3) rarely occurs in photoelectrochemical processes due the electrons of the conduction band are transported *via* an external circuit, by the applied potential (Olea et a., 2021). The generation of holes must occur in a potential range close to the standard redox potential of the ^•^OH (E^o^ = 2.80 eV), that is, the energetic position of the conduction band of the semiconducting material is important (Siahrostami et al., 2017; Olea et al., 2021; Wang et al., 2021). BiOX-based materials meet this requirement, so they have been widely used in photo-and photoelectrocatalysis (Xiong et al., 2020; Han, 2021). The electrons concentrated on the cathode surface induce reduction reactions, essentially reduction of O_2_ present in solution to produce superoxide radicals (O_2_^•−^, another oxidant).

In addition to the surface reactions that occur in the photoelectrodes, the processes that happen at the interface between the semiconductor and the aqueous medium, specifically between the electrode and the supporting electrolyte (Schottky junction), are also important. This interface is capable of modifying the average electron potential (Fermi energy level), which is closer to the valence band in p-type semiconductors (Alulema-Pullupaxi et al., 2021b). This is the case for most semiconductor materials based on BiOXs (Singh, Sharma and Khanuja, 2018). Inside the semiconductor material, is the Space Charge Layer (SCL) where a bending of the valence and conduction bands to different energy levels occurs when the material is irradiated with electromagnetic energy, this is called bending band in Figure 7. This bending results in a strategy to improve the separation of ${\text{e}}_{\text{CB}}^{-}/{\text{h}}_{\text{VB}}^{+}$ pairs and decrease the recombination rate, Figure 7 (Nellist et al., 2016; Kusmierek, 2020; Monllor-Satoca et al., 2020; Peter et al., 2020; Alulema-Pullupaxi et al., 2021a).

Although PEC requires complex systems because of the need for a stable photoelectrode, the application of a bias potential and a light source, it is clear that its advantages outweigh these drawbacks when compared to the degradation process using photocatalysis. In fact, the electrical energy consumed in photoelectrocatalytic degradation is less than that needed for anodic oxidation, which often requires high cell potentials to achieve mineralization (Arotiba et al., 2020).

### 4.2 Environmental Applications of BiOX-Based Materials

Many BiOX-based materials have been developed for environmental remediation applications, and most of these semiconductors have been successful in photocatalytic degradation systems (Huizhong et al., 2008; Chang et al., 2010; Garg et al., 2018). Some authors such as Gao et al. (2021), Wei et al. (2021), and Nava-Núñez et al. (2020) have recently summarized some of the applications of BiOX-based materials in photocatalytic processes. Similarly, Arumugam and Choi (2019) systematically presented the preparation methods and properties of some BiOI-based photocatalysts and their applications in water remediation. Lee et al. (2015) reported the synthesis, characterization, and photocatalytic activity under visible light conditions of some BiOI composites. Further, Liu and Peng (2020) presented the most recent progress in the development of BiOCl photocatalysts.

It is important to note that there are few reports of PEC applications focused on environmental remediation. Nevertheless, some research has focused on the development of photoelectrochemical sensors for photoanodic detection of pollutants (Wang et al., 2020). Similarly, some studies have explored the photoelectrochemical behavior of BiOX-based photoanodes (Poznyak and Kulak, 1990; Kwolek and Szaciłowski, 2013; Stephenson et al., 2018) and photocathodes (Bai et al., 2019), under various photoelectrocatalytic reaction conditions. It should be mentioned that just a few studies have discussed theoretical aspects of the electronic and structural characteristics, in addition the optical properties of the synthesized materials. However, a discussion based on computational calculations could be compared to results from experimental applications and subsequently use to predict the behavior of the semiconductors in the electrochemical cell. In the following, we discuss the most recent results in the development of BiOX-based photoelectrocatalysts applied to the decontamination of aqueous media.

#### 4.2.1BiOCl-Based Materials

As mentioned in the above section, one of the most important properties in photocatalytic activity is the band-gap energy. BiOCl has a band-gap approaching that of TiO_2_ in its anatase phase (∼3.20 eV); therefore, most composites based on this modified first oxide have been produced to improve this feature, as well as its response to visible light. In this regard, Fu et al. (2016) successfully prepared TiO_2-x_/BiOCl heterojunctions (x is the molar ratio of BiOCl to TiO_2_) and examined the photoelectrocatalytic activity in a three-electrode system. TiO_2-x_/BiOCl composites were deposited on an FTO electrode to form the photoanode. Using a 300 W Xe lamp fitted with an AM 1.5 global filter as the light source, they demonstrated that the separation and transport efficiencies of the photogenerated charge carriers were higher for the composite with a 0.3 Bi:Ti molar ratio (TB-0.3) compared to the other photoanodes (TB-0.1 and TB-0.6). The photocurrent of the TB-0.3 photoelectrode increased by the photocurrent density of pure TiO_2_ and BiOCl by 10.9 and 22.21 times, respectively. The increased photocurrent density of this new material indicates that it has a high ability to promote the formation and, transfer of and avoid the recombination of ${\text{e}}_{\text{CB}}^{-}/{\text{h}}_{\text{VB}}^{+}$ pairs (Faraji et al., 2019; Peter et al., 2020). In a more recent study, Mao et al. (2022) prepared a CuO/BiOCl p-n type heterojunction for simultaneous photoelectrochemical detection and photoelectrocatalytic degradation of aflatoxin B1 (AFB1). The modification with CuO nanoparticles improved the responsivity of BiOCl and thus the photocurrent conversion. Using a three-electrode system, they achieved an 81.7% degradation efficiency of a 5.0 μg/L solution of AFB 180 min of reaction. This system thus provides a useful method to control AFB1 contamination in food safety areas specifically.

#### 4.2.2 BiOBr-Based Materials

**FIGURE 8:**
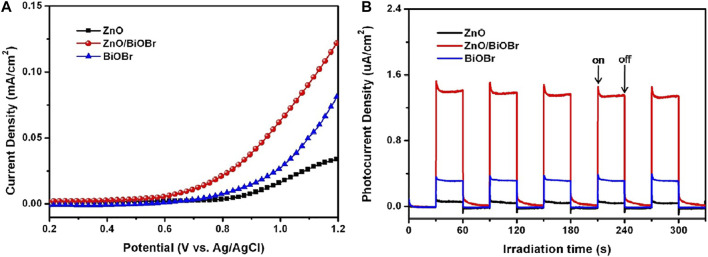
**(A)** Current density versus applied potential curves for ZnO, BiOBr, and the ZnO/BiOBr heterojunction under visible light conditions. **(B)** Photocurrent density response versus time of ZnO, BiOBr, and ZnO/BiOBr photoanodes under visible light irradiation. From reference (Paquin et al., 2015).

Unlike BiOCl materials, bismuth oxybromides have the ability to carry out oxidation-reduction reactions driven by visible light irradiation, because of their slightly narrower band-gap energy (∼2.7 eV). However, their fast charge recombination rate and low redox capacity have driven the construction of new BiOBr-based structures (Guo et al., 2021). In this context, Paquin et al. (2015) synthesized BiOBr nanostructures decorated with vertically aligned ZnO nanorods (ZnO/BiOBr). The decoration constitutes a surface modification resulting in a ZnO termination on the BiOBr surface (Li et al., 2021). In this study, a typical system with three electrodes and a 300 W Xe lamp, the photoelectrochemical behavior of this heterojunction was examined in a 0.5 M Na_2_SO_4_ electrolyte solution; results showed that the optimum content of the raw materials to form BiOBr nanosheets was 0.05 mM [KBr + Bi(NO_3_)_3_] since, at this value, current density reached its maximum. Above this value, aggregates of BiOBr nanoparticles were formed, which led to an increase in the recombination rate of photoexcited ${\text{e}}_{\text{CB}}^{-}/{\text{h}}_{\text{VB}}^{+}$ pairs. Under a potential sweep from 0.2 to 1.2 V vs. Ag/AgCl a photocurrent response of the ZnO/BiOBr photoanode was identified at approximately 0.64 V, a lower value compared to those of ZnO (0.80 V) and BiOBr (0.79 V) (Figure 8A). Additionally, the ZnO/BiOBr photoanode had a higher photocurrent density at lower potential values than the ZnO and BiOBr potentials, indicating a decrease in charge recombination in the vicinity of the flat band potential. Conversely, Figure 8B shows the photocurrent density response versus time of the prepared photoanodes with an open circuit potential. The increasing and decreasing photocurrent indicates that the photoanode’s interface charge transport is very fast, which is attributed to the electron transfer directly from the aligned ZnO nanorods. In addition, the high photocurrent density indicates the effective and rapid separation of charge carriers.

Similarly, Liu et al. (2018) combined the photoelectrochemical degradation of tetracycline using BiOBr as a photoanode and simultaneous CO_2_ reduction with a CuO cathode. After applying a potential sweep from 0.6 to 0.8 V vs. Ag/AgCl, 68% of the drug was degraded in 2.5 h of reaction under illumination from a 300 W Xe lamp equipped with a 420 nm cutoff filter, using Pt as a counter electrode. When coupled, CuO as a cathode achieved 80% degradation of the tetracycline at 0.7 V in a photoelectrocatalytic system under the aforementioned conditions. These results demonstrate that there is a significant synergy between the photoelectrocatalytic degradation of tetracycline and CO_2_ reduction in the BiOBr/CuO system. In another study, Ling et al. (2020) successfully prepared BiOBr nanosheet arrays (NSAs) using a solvothermal method, and examined their photoelectrocatalytic ability in the degradation of some organic pollutants. Linear scanning voltammetry (LSV) results showed a photocurrent response of the BiOBr NSA-160 photoanode (160 indicates the temperature at which the electrode was prepared) at a bias voltage of 1.4 V under dark conditions. When irradiated with visible light, the photocurrent intensity increased until the bias voltage exceeded 0.9 V. Thus, the increased voltage causes the photoexcited electrons to be transferred to the external circuit, thereby improving the separation efficiency of ${\text{e}}_{\text{CB}}^{-}/{\text{h}}_{\text{VB}}^{+}$ pairs (Jeon et al., 2018; Wang et al., 2018). The photoelectrocatalytic activity was evaluated in the degradation of ciprofloxacin (CIP), 91.4% degradation was achieved at the end of 180 min, which was reached once the applied bias voltage was increased up to 0.9 V. The degradation efficiency of CIP increased only slightly once the bias voltage exceeded 0.9 V and decreased when the voltage exceeded 1.8 V. This effect occurs because proper biasing can effectively transfer the photogenerated electrons.

**FIGURE 9:**
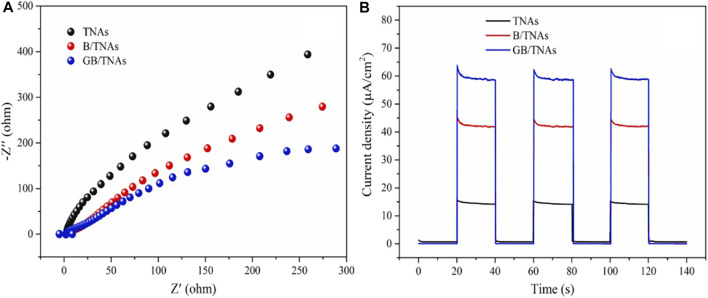
**(A)** Nyquist plot for TNAs, B/TNAs, and GB/TNAs photoelectrodes. **(B)** Photocurrent responses of TNAs, B/TNAs, and GB/TNAs photoanodes. From reference (Ma et al., 2021).

In a more recent study, using an electrodeposition method Ma et al. (2021) synthesized a ternary heterostructure of reduced graphene oxide (rGO)/BiOBr/TiO_2_ nanotube arrays (GB/TNAs) for the photoelectrocatalytic degradation of *p*-chloronitrobenzene, the charge transfer kinetics were analyzed *via* electrochemical impedance spectroscopy (EIS). The Nyquist plot in Figure 9A shows that the GB/TNAs photoanode has the lowest semicircle signal with respect to the other analyzed photoelectrodes, TNAs (TiO_2_ nanotube arrays) and B/TNAs (BiOBr/TiO_2_ nanotubes). This indicates that the structure of BiOBr and rGO promoted the transfer of the TNAs charge carriers with reduced resistance. On the other hand, Figure 9B shows the photocurrent response over time under cyclic visible light irradiation in an on/off system. The GB/TNAs electrode stabilized at approximately 60 μA/cm^2^, which was 3.75 and 1.43 times higher than that of TNAs and B/TNAs, respectively. This indicates that the GB/TNAs heterojunction has a higher photogenerated charge separation ability. The highest degradation efficiency of a 0.1 mM sample of *p*-chloronitrobenzene was achieved after 6 h of visible light irradiation (300 W Xe lamp), reaching a value of 86.8% using the GB/TNAs photoanode. This value was higher than the efficiencies reported for the B/TNAs and TNAs photoanodes (72.0 and 41.5%, respectively), indicates that introducing of rGO to the semiconductor structure favors the photoelectrocatalytic activity of the system due to its excellent conductivity. Additionally, the pseudo-first order reaction constants corresponding to the degradation process were reported: the constants increased from 0.0037 to 0.0055 min^−1^ when a potential sweep from 0 to 1.0 V was applied and decreased when the potential exceeded 1.5 V. Importantly, photoelectrocatalytic activity was limited by the surface structure of the semiconducting materials serving as photoanodes, in addition to their optical properties and charge separation ability to prevent recombination (Ma et al., 2021). In this context, structural analysis of the photoelectrode surface showed that the BiOBr/TNAs structure was compact, which contributed to better visible light collection and higher charge separation efficiency compared to that of pure TNAs.

#### 4.2.3 BiOI-Based Materials

BiOI is a p-type semiconductor, with an even narrower bandgap energy than BiOCl and BiOBr (approximately 1.70 eV), and thus, has been considered one of the most promising materials for environmental remediation in both photocatalytic and photoelectrocatalytic systems (Zhang et al., 2008; Hu et al., 2014; Huang et al., 2017). Recently, numerous studies have focused on the design of new BiOI-based materials to treat different pollutants through photocatalytic processes, in which BiOI/TiO_2_ heterojunctions (Liao et al., 2021) and Z-type Fe_3_O_4_/BiOCl/BiOI heterostructures (Dang et al., 2022) are included. Additionally, semiconductors based on non-stoichiometric bismuth oxyiodides (Bi_x_O_y_I_z_), also called bismuth-rich bismuth oxyiodides, whose structures exhibit improved stability and whose band structures are more suitable for photocatalytic processes has been reported (Liu and Zhang, 2014; Jin et al., 2017). In addition, multi-walled carbon nanotube/Bi_4_O_5_I_2_ (Gao et al., 2022), La_2_Ti_2_O_7_/Bi_5_O_7_I (Zhu et al., 2022), 2D/3D BiOCl/Bi_5_O_7_I heterojunctions (Huang et al., 2022), and Bi_7_O_9_I_3_/g-C_3_N_4_/Bi_3_O_4_Cl (Yuan et al., 2022) heterostructures have been reported.

**FIGURE 10:**
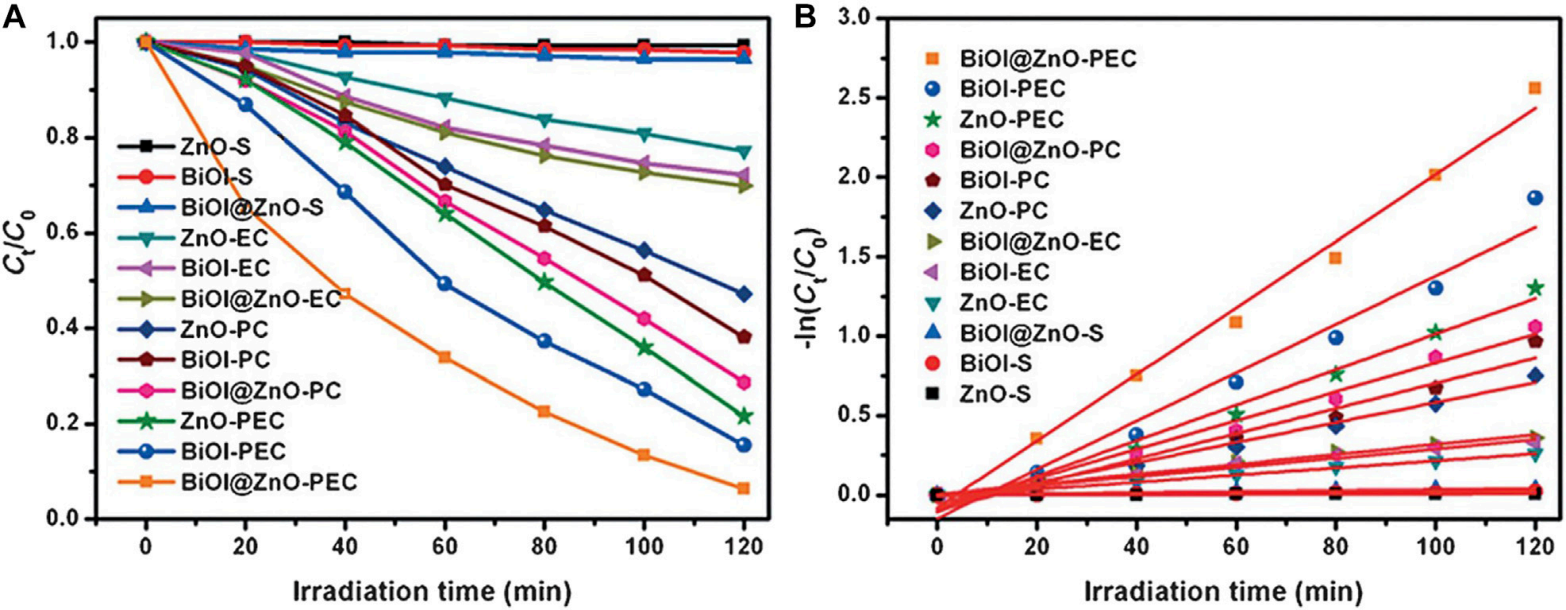
**(A)** Relationship of Congo red degradation efficiencies of EC (electrocatalysis), PC (photoelectrocatalysis), S (submerged sample without illumination) catalytic processes with respect to ZnO, BiOI, and ZnO/BiOI. **(B)** -ln (C_t_/C_0_) vs. visible light irradiation time. From reference (Kuang et al., 2015).

In the context of PEC, BiOI-based materials have been developed to improve photoanode stability and, charge separation ability and decrease the recombination rate (Guo et al., 2019). In one related study, Kuang et al. (2015) prepared a p-n type BiOI/ZnO heterojunction using a solvothermal method for the efficient degradation of Congo red (an organic dye). In a three-electrode system and with a 0.5 M Na_2_SO_4_ solution, the photoelectrochemical behavior of this composite was investigated. The maximum current density identified at 1.2 V was 0.20 mA/cm^2^ which corresponds to a 2.22-fold increase with respect to ZnO and 1.43-fold increase with respect to the BiOI film. After 2 h of reaction, the BiOI/ZnO photoanode had the highest Congo red degradation efficiency, reaching a value of 93.66%. Figure 10A shows the comparative relationship of the various degradation processes carried out for this dye. The electrocatalytic and photocatalytic processes reveal a lower degradation efficiency compared to the photoelectrocatalytic process (PEC). Similarly, as shown in Figure 10B the kinetic constant of the pseudo-first order reaction is significantly higher in the photoelectrocatalytic process using the BiOI/ZnO material in comparison to the rest of the procedures and materials.

**FIGURE 11:**
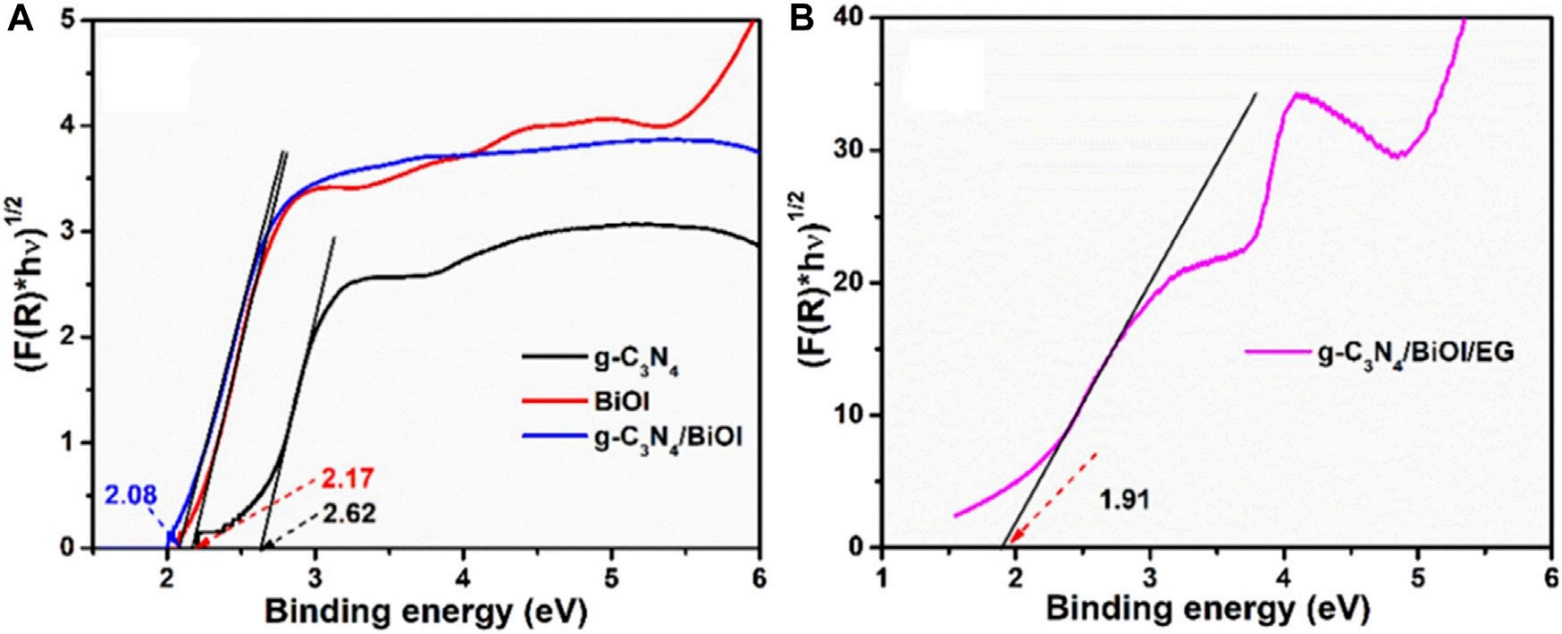
**(A)** Tauc diagrams of g-C_3_N_4_, BiOI, g-C_3_N_4_/BiOI, and **(B)** g-C_3_N_4_/BiOI/EG. From reference (Mafa et al., 2019).

Similarly, Sun et al. (2017) took advantage of the high capacity of CuI as a positive gap carrier and the excellent visible light absorption of BiOI to design a CuI/BiOI heterostructure with exceptional photoelectrocatalytic properties. To demonstrate its degradation capability, Pt nanoparticles were deposited on the composite’s surface, and used to degraded methanol and methylene blue (an organic dye) in alkaline solution under dark and visible light. Results showed the optimum amount by weight of Pt nanostructures on the photoanode surface was 10%, above this value, the Pt nanoparticles aggregated with each other, resulting in a high charge density, which decreasing the catalytic ability of the structure. In a typical three-electrode system, using a saturated calomel electrode (SCE) as a reference, 87% of a 10 mg/L solution of methylene blue with Na_2_SO_4_ as supporting electrolyte was degraded. Compared to the efficiencies obtained using pure BiOI and CuI (68 and 8%, respectively), the improvement of the photoelectrocatalytic activity of BiOI is attributed to the introduction of CuI as a promoter of charge separation. Some of the most relevant applications reported thus far focus on the photoelectrocatalytic decomposition of active pharmaceutical ingredients. In this field, Mafa et al. (2019) synthesized a photoanode based on a p-n g-C_3_N_4_/BiOI/EG type heterojunction (EG: exfoliated graphite), for the photoelectrocatalytic degradation of sulfamethoxazole (an antibiotic); an approximate removal efficiency of 88% was achieved at a maximum current density of 5 mA/cm^2^ and a pH of 6.23. They also reported the estimated band-gap energy of the synthesized materials. Figures 11A,B show the Tauc plots obtained for the materials, which demonstrate the decreased band-gap energy of the g-C_3_N_4_/BiOI/EG composite (∼1.91 eV).

In another study, Liu et al. (2019) highlighted the stability of the electrodeposition-prepared BiOI/BiPO_4_/FTO photoanode in the photoelectrocatalytic degradation of tetracycline in an aqueous medium. XRD results before and after the cycling photoelectrocatalytic tests showed that the BiOI/BiPO_4_/FTO photoelectrode had excellent stability to degrade this pharmaceutical pollutant. The degradation rate was not affected after four 16 h degradation cycles. Thus, it can be considered a material with excellent characteristics for decontaminating aqueous media in photoelectrocatalytic systems.

In addition to requiring a stable photoanode with good effectiveness against visible light, photoelectrocatalytic degradation efficiency is further limited by the reaction pathway the pollutant molecule follows to mineralization or, in other cases, to simpler substances (Yang et al., 2018; Ahmad et al., 2021). Orimolade et al. (2019) reported degradation efficiencies of acetaminophen (68%), CIP (62%), and orange II (85%) for a photoelectrocatalytic system using a BiVO_4_/BiOI composite as the photoanode, they also determined the charge transfer mechanism in the photoelectrochemical system. During the formation of the internal electric field when BiVO_4_ and BiOI are in contact, electrons are able to migrate until the Fermi energy level are equivalent between the two semiconducting materials. After this process, the band energy edges of the modified bismuth oxides are adjusted producing a potential difference between these two materials; that is, the electrons accumulated in the conduction band of BiOI migrate towards the conduction band of BiOV_4_ and likewise the positive vacancies (photogenerated holes) (Orimolade et al., 2019). The migration direction of the photogenerated electrons and holes into the conduction and valence bands depends on the type of heterojunction formed between the composing semiconductors (Y. Li et al., 2021). In this way, the composite achieves a better charge separation and thus a higher degradation efficiency compared to individual materials. This is because, there are more holes able to oxidize the contaminant molecules or produce hydroxyl radicals (strong oxidants) when they react with the water, and superoxide radicals when the electrons on the cathode surface reduce the oxygen in the solution (Younis et al., 2020; Garcia-Segura et al., 2021).

Definitely, Table 1 summarizes the most recent applications of BiOX-based photoanodes prepared by various synthesis methods, intended for the degradation of organic pollutants in aqueous media. It also includes the experimental conditions of the photoelectrochemical systems used for the visible light-driven degradation reactions and the reported degradation efficiencies for some organic pollutants, particularly highlighting active pharmaceutical ingredients, including antibiotics and analgesics, organic dyes, and some phenol derivatives.

**TABLE 1:** Characteristics of BiOX-based materials in the photoelectrocatalytic degradation of some organic pollutants.

Semiconductor material (photoanode)	Synthesis/Preparation method	Pollutant type	Pollutant	Experimental conditions	Degradation efficiency (%)	References
g-C_3_N_4_/BiOI/EG	Ultrasound assisted	Antibiotic	Sulfamethoxazole (SMX)	CE: Pt wire RE: Ag/AgCl (3 M KCl) t: 30 min; pH: 6.23 CD: 5 mA/cm^2^; HAL-320 (Xe lamp 300-W)	88.0	Mafa et al. (2019)
CuI/BiOI	Hydrothermal-ultrasound Assisted	Organic dye	Methylene blue (MB)	CE: Pt wire; RE: SCE t: 2 h; V: 20 ml C_o_: 10 mg/L Xe lamp 300-W	87.0	Sun et al. (2017)
ZnO/BiOBr	Solvothermal	Organic dye	Rhodamine B (RhB)	CE: Pt sheet (1 cm^2^) RE: Ag/AgCl t: 100 min; V:50 ml; C: 0.1 mM; 0.1 M Na_2_SO_4_; Xe lamp 300-W	95.4	Paquin et al. (2015)
BiOBr/TiO_2_ (B/TNAs)	Adsorption and successive ionic layer reaction (SILAR)	Phenolic derivate	*p-*chloronitrobenzene (p-CNB)	CE: Pt foil, t: 6 h; V: 50 ml; C: 0.1 mM	72.0 ± 3.2	Ma et al. (2021)
Graphene oxide/BiOBr/TiO_2_ (GB/TNAs)	Electrodeposition				86.8 ± 3.6	Ma et al. (2021)
ITO glass/BiOBr NSA-160	Solvothermal	Antibiotic	Ciprofloxacin (CIP)	CE: Pt plate RE: Ag/AgCl (3 M KCl) t: 180 min; C: 15 mg/L; 0.1 M Na_2_SO_4_, CD: 69 μAcm^−2^ Xe lamp 500-W	91.4	Ling, Dai and Zhou, (2020)
		Organic dye	Rhodamine B (RhB)	CE: Pt plate RE: Ag/AgCl (3 M KCl) t: 90 min; Xe lamp 500-W	94.3	Ling, Dai and Zhou, (2020)
		Antibiotic	Tetracycline	CE: Pt plate RE: Ag/AgCl (3 M KCl) t: 180 min; C: 15 mg/L; 0.1 M Na_2_SO_4_, Xe lamp 500-W	93.2	Ling, Dai and Zhou, (2020)
		Organic derivate	Bisphenol A	CE: Pt plate RE: Ag/AgCl (3 M KCl) t: 180 min; C: 15 mg/L; 0.1 M Na_2_SO_4_; Xe lamp 500-W	67.5	Ling, Dai and Zhou, (2020)
		Analgesic	Acetaminophen	CE: Pt foil RE: Ag/AgCl (3 M KCl) t: 2 h; C: 10 mg/L; 0.1 M Na_2_SO_4_; V: 50 ml Xe lamp 100-W	68.0	Orimolade et al. (2019)
FTO/BiVO_4_/BiOI	Electrodeposition	Antibiotic	Ciprofloxacin (CIP)	CE: Pt foil RE: Ag/AgCl (3 M KCl) t: 2 h; C: 10 mg/L, 0.1 M Na_2_SO_4_; V: 50 ml Xe lamp 100-W	62.0	Orimolade et al. (2019)
		Organic dye	Orange (II)	CE: Pt foil RE: Ag/AgCl (3 M KCl) t: 2 h; 0.1 M Na_2_SO_4_ Xe lamp 100-W	85.0	Orimolade et al. (2019)
Bi_2_O_3_-BiOI	Simple immersion	Organic derivate	Phenol	CE: Ti; pH: 6.2 V: 50 ml; C: 10 mg/L, 0.2 M Na_2_SO_4_ Xe lamp 500-W	—	Cong et al. (2017)
CuO/BiOCl/ITO	*In-situ* growth	Organic derivate	Aflatoxin B1 (AFB1)	CE: Pt wire RE: Ag/AgCl saturado t: 180 min; C: 5 μg/L PEAC 200 A PEC reaction instrument	81.7	Mao et al. (2022)
BiOBr/FTO	Solvothermal	Antibiotic	Tetracycline	CE: CuO RE: Ag/AgCl t: 2.5 h; V: 80 ml; C: 10 ppm, 0.1 M Na_2_SO_4_; 0.1 M KHCO_3_ Xe lamp 300-W	80.0	Liu et al. (2018)
BiOI/BiPO_4_/FTO	Electrodeposition	Antibiotic	Tetracycline	CE: Pt wire RE: SCE V: 100 ml; C: 10 ppm 0.1 M Na_2_SO_4_ Xe lamp 500-W	77.0	Liu et al. (2019)
FTO/TiO_2-x_/BiOCl	Ultrasound Assisted	Organic dye	Rhodamine B (RhB)	CE: Pt wire RE: Ag/AgCl 0.5 M Na_2_SO_4_ Xe lamp 300-W	—	Fu et al. (2016)
FTO/BiOI/ZnO	Electrodeposition	Organic dye	Congo red (CR)	CE: Pt sheet RE: Ag/AgCl t: 2 h V: 60 ml; C: 0.1 mM 0.1 M Na_2_SO_4_ Xe lamp 300-W	93.6	Kuang et al. (2015)
BiOI/GH/FTO	Electrodeposition	Organic derivate	Phenol (Ph-OH)	CE: Pt wire RE: SCE t: 5 h; C: 5 ppm 0.1 M Na_2_SO_4_ Xe lamp 500 mW/cm^2^	83.0	Chen et al. (2018)

CE, Counter electrode; RE, Reference electrode;

## 5 Conclusion and Future Challenges

In response to the need to develop new economical, efficient, and environmentally friendly technologies for pollutant degradation interest has grown in developing innovative, low-cost semiconductor materials able to degrade pollutants present in different ecosystems at low concentrations. The present review has focused on analyzing the most recent research on the properties of BiOX (X = Cl, Br, and I)—based materials and their ability to degrade organic compounds in aqueous media by means of PEC; this relatively new technology, which is undergoing constant innovation, enhances the characteristics of heterogeneous photocatalysis by applying advanced electrochemical oxidation. In addition, the optimal experimental conditions of each photoelectrocatalytic system and the degradation efficiencies for some organic pollutants were presented. The optical and structural properties that demonstrate the high capacity of these oxides to capture and harness visible light energy to promote important oxidation-reduction reactions in a photoelectrochemical process were also addressed. Finally, the advantages and disadvantages of the photoelectrocatalytic mechanism were discussed, with an emphasis on the use of BiOX-based materials as photoanodes.

The present review highlights the countless challenges and possibilities for improving the characteristics and properties of BiOX-based materials, and other semiconductors. It also focuses on the systematization of new technologies that take advantage of the performance of these semiconductors, for example by directly utilizing solar energy instead of light sources simulating such energy, to make these procedures not only more efficient but also environmentally and economically advantageous. Therefore, great efforts must be made to limit the economic and energy costs in the design and construction of materials and technologies based on advanced electrochemical processes.

Another interesting challenge with BiOX-based photocatalysts that only a few studies have reported with is the optimization of photochemical and photoelectrochemical processes by coupling new systems—that is, two or more processes occurring simultaneously in the electrochemical cell—to gain complete control of the kinetics, thermodynamics and efficiency of the processes. Ultimately, all efforts to improve existing or generate new and innovative technologies should center on readying these systems to be engineered and introducing these technologies and materials in real water treatment plans that prioritize pollutant removal to have an actual impact on the environment.
